# Personalized volume de-escalated elective nodal irradiation in oropharyngeal squamous cell carcinoma (DeEscO): a study protocol

**DOI:** 10.1016/j.ctro.2026.101182

**Published:** 2026-05-12

**Authors:** Esmée L. Looman, Yoel Pérez Haas, Roman Ludwig, Debra Lauer, Grégoire B. Morand, Matthias Guckenberger, Olgun Elicin, Roland Giger, Sébastien Tran, Francesco Martucci, Sarah Melab-Belkhodja, Oliver Riesterer, Stephan Benke-Bruderer, Jan Unkelbach, Panagiotis Balermpas

**Affiliations:** a Department of Radiation Oncology, University Hospital Zurich, Zurich, Switzerland; b University of Zurich, Zurich, Switzerland; c Department of Otorhinolaryngology, University Hospital Zurich, Zurich, Switzerland; d Department of Radiation Oncology, Inselspital, Bern University Hospital and University of Bern, Bern, Switzerland; e Department of Otorhinolaryngology, Head and Neck Surgery, Inselspital, Bern University Hospital and University of Bern, Bern, Switzerland; f Department of Radiation Oncology, University Hospital Geneva, Geneva, Switzerland; g Department of Radiation Oncology, Ospedale Regionale di Bellinzona, Bellinzona, Switzerland; h Department of Radiation Oncology, Réseau hospitalier neuchâtelois, Neuchâtel, Switzerland; i Department of Radiation Oncology, Kantonsspital Aarau, Aarau, Switzerland; j Clinical Trials Center, University Hospital Zurich, Zurich, Switzerland

**Keywords:** Head and neck cancer, Oropharyngeal squamous cell carcinoma, Radiotherapy, Elective nodal irradiation, Clinical target volume, De-escalation

## Abstract

**Background:**

Definitive (chemo)radiotherapy of oropharyngeal squamous cell carcinoma (SCC) consists of treatment of the macroscopic tumor and prophylactic irradiation of the lymphatic drainage system: the elective clinical target volume (CTV). In this study, we reduce the elective CTV based on the personalized factors T-stage, clinical lymphatic involvement, and lateralization of the primary tumor.

**Methods:**

The primary objective is to evaluate efficacy and safety of an individualized de-escalation of elective node irradiation volumes. We collected a multi-institutional dataset of 598 oropharyngeal SCC patients in whom detailed lymph node involvement was reported. Based on the data, we developed a model of lymphatic tumor progression to estimate the probability of occult metastases in the clinically negative lymph node levels (LNLs). The dataset can be visualized on https://lyprox.org/. Based on this model and clinical judgement, we created tables that define the personalized elective CTV for different combinations of T-stage, midline extension and clinical LNL involvement. The primary endpoint of this multicentric, prospective, single-arm trial is the rate of out-of-field nodal recurrences after 2 years. The study will be conducted in 6 centers across Switzerland and 120 patients will be enrolled.

**Discussion:**

This study sets out to personalize the elective CTV in oropharyngeal SCC patients, with the goal to de-escalate radiotherapy for patients and herewith improve therapy-associated toxicity and quality of life. The results of this study may be used to personalize treatment in oropharyngeal SCC patients.

**Trial registration:**

The trial was registered on ClinicalTrials.gov NCT06563362, on 20/08/2024. Ethical approval was obtained on 21/01/2025, BASEC-Nr 2024-01936. Protocol Version 2.1, 12/05/2025.

## Highlights

• Multicenter trial for individualized volume de-escalation in oropharyngeal SCC.
• Reduced elective CTV based on T-stage, lymphatic involvement, and lateralization.
• No contralateral nodal irradiation for completely lateralized tumors.
• Only irradiating LNL IV in case of LNL III involvement.
• Sparing of LNLs I and V in many patients.

## Introduction

Curative intended treatment of squamous cell carcinoma (SCC) of the oropharynx can consist of surgery, (chemo)radiotherapy, or a combination of both. When treated with radiation, a large part of the lymphatic drainage system of the neck which is at risk of harboring occult metastases, is irradiated − the “elective clinical target volume (CTV)” [1], [2], [3]. The elective CTV is currently based on clinical recommendations, but there is only limited quantitative data available on (occult) lymphatic spread and the required size of the elective CTV [3], [4], [5], especially with regard to further personalization.

Elective irradiation of the neck lymph node levels (LNL) is associated with early and late toxicities, which potentially lead to hospitalization or long-term symptoms with subsequent life-quality impairment [6], [7], [8], [9], [10], [11]. In addition, there is increasing interest in possible immune-sparing effects of radiotherapy de-escalation. Irradiation of the lymph nodes and lymph drainage system is associated with the development of lymphopenia [12]. By volume de-escalation, the lymphatic system could be spared more, potentially leading to improved antigen-priming, less lymphopenia and better survival outcomes [13], [14], [15], [16], [17].

Several observations suggest that there is potential for safely reducing the elective CTV without substantially increasing the risk of regional failure. There is low incidence of treatment failure and out-of-field lymph node metastases after standard-of-care (chemo)radiotherapy in oropharyngeal SCC. Leeman et al. showed a local treatment failure at 5 years of 4.2%, with 2.9% regional nodal failure and no marginal or isolated out-of-radiation-field failure [18]. Similar results were found by Van den Bosch et al., who found a 2-year recurrence rate in electively irradiated lymph node regions of 5.2% [19]. Several trials applying reduced elective CTVs have reported low rates of N-site recurrences in unirradiated levels. This includes studies on unilateral neck irradiation for lateralized tumors [20], [21], [22] as well as the EVADER trial [23]. Many de-escalation studies have focused on HPV-positive tumors because of the favorable prognosis or found better results for the patients with HPV-positive tumors when de-escalating treatment [23], [24], [25], [26], [27], [28].

We developed a statistical model to estimate the patients’ individual risk of occult lymph node metastases in clinically negative LNLs in newly diagnosed oropharyngeal SCC patients, based on the patient’s location of clinically detected metastases, T-stage, and lateralization of the primary tumor [29], [30], [31], [32]. The elective CTV is subsequently defined based on a patient’s individualized risk such that the overall risk of occult lymph node metastases in all non-irradiated LNLs (out-of-field) is estimated to be below 10%. This leads to substantially smaller elective CTVs compared to current guidelines [3].

The aim of this phase II clinical trial is to determine the efficacy and safety of the use of a personalized volume de-escalated elective nodal CTV in oropharyngeal SCC patients treated with primary (chemo)radiotherapy.

### Previous work leading to the trial design

We collected a multi-institutional dataset of 598 oropharyngeal SCC patients in whom the detailed patterns of lymph node involvement are reported. For each patient, lymph node involvement was recorded for each individual LNL based on the available diagnostic modality. Pathology results after neck dissection was reported for the subset of surgically treated patients. In addition, primary tumor location, lateralization, HPV-status, and T-stage is reported among other patient and tumor characteristics. The dataset is publicly available [33], [34], [35]. The publicly available online platform LyProX.org was developed to visualize published datasets in an intuitive and interactive manner.

To define the elective nodal CTV, we are interested in the conditional probability that a clinically negative LNL harbors occult metastases, given the primary tumor characteristics and the location of clinically detected lymph node metastases. This conditional probability of occult disease depends on 1) the probability of the tumor to spread to the LNL of interest, and 2) the sensitivity and specificity of clinical diagnostic methods. To combine both aspects, we developed a comprehensive statistical model to predict the probability of occult disease per LNL using a Hidden Markov model (HMM). In this model, the state of involvement of a LNL is described by a latent binary random variable, which indicates if the LNL is healthy or harbors metastases (including occult metastases). The state of lymph node involvement of a patient is then described by a vector of binary random variables. Each LNL is further associated with an observed state, another binary random variable that indicates if a LNL is clinically diagnosed as involved. The HMM is used to describe the spread from the primary tumor to the LNLs and progression from an involved LNL to a downstream LNL. The model is parameterized using a directed graph where the arcs represent the possible spread to and between LNLs, resembling the anatomically defined lymph drainage of the neck. The main model parameters are the probabilities of spread associated with each arc. The latent true state of involvement and the clinically observed involvement are linked via sensitivity and specificity of imaging. Thus, we can compute the likelihood of the observed dataset of 598 patients given the model parameters. We use Markov Chain Monte Carlo (MCMC) sampling to draw these parameters from the posterior distribution, comprised of the likelihood and a uniform prior. This approach naturally provides uncertainty bounds for the model's parameters and consequently also for the predicted risk of occult involvement. Model training is performed based on a consensus decision on the most likely state of involvement given all diagnostic modalities available for each patient and LNL in the dataset, which is assumed to represent the true latent state of involvement. Sensitivities and specificities are not learned, but taken from literature values, originating from the comparison of clinical and pathological involvement of patients receiving neck dissection. For risk calculations we set specificity to 1 and sensitivity to 0.81. This corresponds to the literature values for sensitivity of CT and MRI reported by De Bondt et al. [36]. A specificity of 1 corresponds to the assumption that clinically involved LNLs truly harbor metastases and do not represent false positives. A detailed description of the methodology can be found in the respective publications [29], [30], [31], [37]. The model presented in [31] is representative for the model underlying the design of the DeEscO trial, despite not being identical in all details.

The trained model can then be used for predicting the probability of occult involvement per LNL for individual patients. The patient characteristics that are input to the model are 1) clinical involvement per LNL, 2) early (T1/T2) versus advanced (T3/T4) T-stage, and 3) lateralization of the primary tumor. In addition, we considered central tumors, where there is no clear origin of the primary tumor lateral to the midline. For the common states of the primary tumor and clinical lymph node involvement (the patients that occurred at least once in the dataset), the model was used to predict the risk of occult disease in the clinically negative LNLs. To define the elective nodal CTV (referred to as CTV-3 below), the clinically negative LNLs are ranked according to their risk of occult involvement. The LNLs with the highest risk are included in the CTV-3 until the cumulative risk of involvement in all remaining LNLs is below 10% for 95% of the model parameter samples generated through MCMC. As a final measure of quality assurance, the CTV-3s constructed in this way have been discussed individually by the investigators, to ensure consistency with data and clinical judgement and experience (Table 1, Supplementary Tables 1–5).

**Table 1:** General rules.

**General rules**	
**1**	Include all involved lymph node levels (LNLs) in the CTV-3.
**2**	Ipsilateral level II (including IIa and IIb) is always irradiated.
**3**	Level Ia is never included in the CTV-3 unless clinically involved. In case of involvement of level Ia, the study PI at USZ must be contacted for a treatment decision.
**4**	If level IV is involved, the entire level (both IVa and IVb) is included in the CTV-3. If level IV is not involved, level IVb is never included in the CTV-3.
**5**	If level V is irradiated according to Supplementary Tables 1–5, levels Va and Vb are irradiated. Level Vc is irradiated if level Vb is involved.
**6**	Level VII is never included in the CTV-3 unless clinically involved. In case of level VIIa involvement, both level VIIa and VIIb are included in the CTV-3. In case of level VIIb involvement, only level VIIb (not VIIa) is included in the CTV-3.
**7**	If a level that is not irradiated according to Supplementary Tables 1–5 but partially overlaps with PTV-1, PTV-2, or PTV-3, then only the overlapping part of the level will be irradiated as part of the respective PTV. The non-overlapping part is not to be included in the CTV-3.

The supplementary tables cover the most commonly observed cases in our database (85% of the cases in our database) and a select number of plausible cases. The investigators at University Hospital Zurich have a table with all (theoretically) possible cases and their treatment recommendations available.

## Design

### Treatment description

The DeEscO study will be conducted as a prospective, multicenter, open label, single-arm trial at six centers in Switzerland (University Hospital Zurich, Bern University Hospital, University Hospital Geneva, Cantonal Hospital Aarau, Cantonal Hospital Bellinzona, and Réseau Hospitalier Neuchâtelois).

The study aims to recruit 120 oropharyngeal SCC patients treated with definitive (chemo)radiotherapy with curative intent.

The study includes patients with newly diagnosed (no pre-treatment) SCC of the oropharynx (ICD-10 codes C01, C09, C10), T1-4, N0-3, irrespective of p16-status. Treatment with definitive (chemo)radiotherapy is planned, including elective irradiation of the cervical lymph nodes. Patients need to have an ECOG performance score ≤ 2, have a consultation with physical examination by a head and neck surgeon, radiation oncologist or medical oncologist within 30 days prior to registration and patients should have received staging with FDG-PET/CT prior to registration. In case of inability to perform or contra-indications, at least a contrast-enhanced MRI is obligatory.

Patients are excluded in case the tumor extends from the oropharynx into other levels, such as the oral cavity, naso- or hypopharynx, or if patients are diagnosed with distant metastases. Previous surgery, chemotherapy or radiotherapy treatment in the head and neck area is not allowed, except for singular lymph node dissections for diagnostic purposes or treatment of Tis-T2 glottic laryngeal carcinoma without treatment of the lymphatic drainage area.

Patients will receive treatment with definitive radiotherapy and if indicated, standard-of-care chemotherapy. Dose prescription and treatment of the primary tumor is performed according to standard-of-care. The study-specific intervention is the personalized definition of the elective CTV as described below.

Radiation volumes and doses are based on Nutting et al. (Lancet Oncology, 2023) [38] and the DAHANCA 2020 guidelines [39].

The radiotherapy volumes will be defined as follows:

• GTV-T: Macroscopic gross tumor in T site
• GTV-N: Macroscopic tumor in N site(s)
• CTV-1: 5 mm expansion of the combined GTV (GTV-T and GTV-N), corrected for anatomical barriers.
• CTV-2: 10 mm expansion of the combined GTV, corrected for anatomical barriers.
• CTV-3: Defined as the elective target volume. LNLs are to be delineated according to Grégoire et al. [1] The LNLs to include in the CTV-3 are defined in Tables 1 and Supplementary Tables 1–5.
• For expansion from CTV to PTV, an expansion of 3–5 mm – to the discretion of the participating center – is used. All PTVs should be cropped to the skin.

### Radiotherapy doses

The following physical radiotherapy doses will be applied to the target volumes:

• CTV-1/PTV-1: 65–70 Gy
• Optional: CTV-2/PTV-2: 60–66 Gy
• CTV-3/PTV-3: 50–56 Gy

Radiotherapy will be given as simultaneous integrated boost (SIB) in 30–35 fractions of 1.8–2.2 Gy (in CTV-1 and CTV-2) and 1.47–1.8 Gy (in CTV-3) over 6–7 weeks – to discretion of the treating participating center. PTV-1 and PTV-2 may receive the same radiotherapy dose (as described e.g. in Nutting et al. – in this case there would be no “intermediate dose”/“intermediate risk volume”), or the PTV-2 can be omitted, if a 10 mm expansion of the GTV is included in the target volume CTV-3.

### Personalized elective nodal CTV de-escalation

**Fig. 1:**
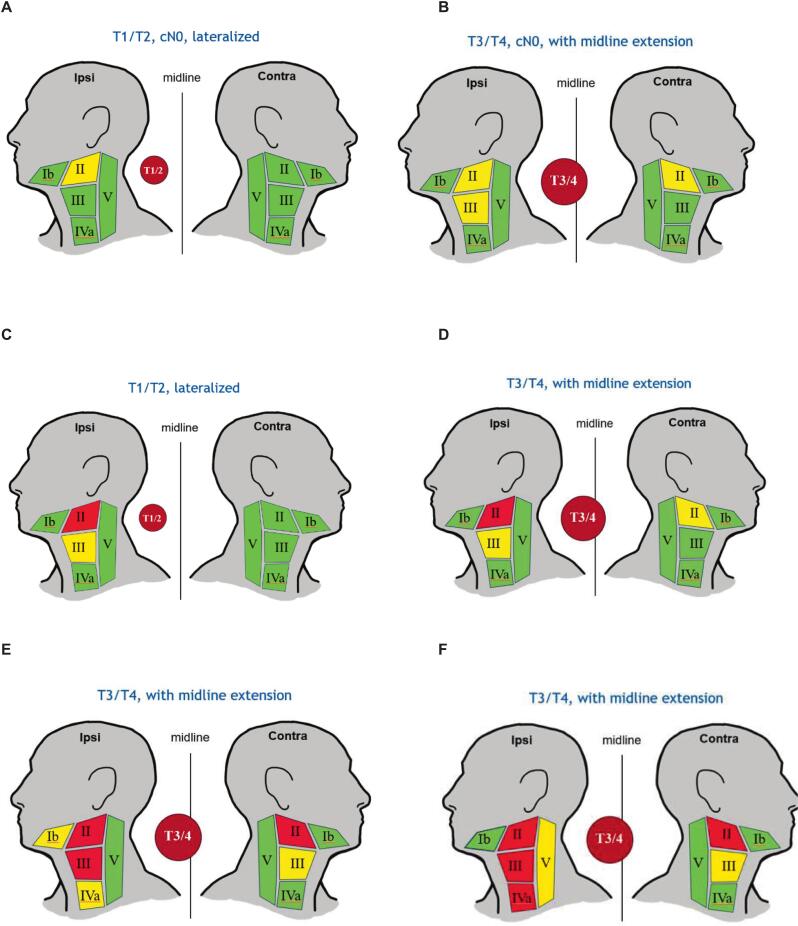
**A-F. Schematic examples of the personalized elective nodal CTV de-escalation.** Schematic examples of the personalized elective nodal clinical target volume (CTV) de-escalation. Green illustrates unirradiated lymph node levels (LNLs), yellow illustrates irradiated LNLs (part of the elective CTV), red illustrates LNLs with macroscopic lymph node metastases. **Fig. 1A** shows a situation where a patient is diagnosed with a T1/T2-stage tumor that does not cross the midline, without any lymph node metastases. In this case, in the trial only ipsilateral level II will be irradiated. **Fig. 1B** shows a patient diagnosed with a T3/T4-stage tumor that crosses the midline, without any lymph node metastases. In this case, LNLs II and III on the ipsilateral side and level II on the contralateral side will be irradiated. **Fig. 1C** shows a patient with a lateralized T1/T2-tumor and macroscopic lymph node metastases in ipsilateral level II. Here, LNLs II and III on the ipsilateral side will be irradiated, no levels on the contralateral side will be irradiated. **Fig. 1D** shows a patient with a T3/T4-tumor with midline extension and macroscopic lymph node metastases in ipsilateral level II. In this case, LNLs II and III on the ipsilateral side and level II on the contralateral side will be irradiated. **Fig. 1E** shows a patient with a T3/T4-tumor, with midline extension, and macroscopic lymph node metastases in ipsilateral levels II and III and contralateral level II. In this case, levels Ib-IVa on the ipsilateral side and levels II and III on the contralateral side will be irradiated. **Fig. 1F** shows a T3/T4-tumor with midline extension diagnosed with macroscopic lymph node metastases in ipsilateral levels II, III and IVa and contralateral level II. Here, ipsilateral levels II-V and contralateral levels II and III will be irradiated. (For interpretation of the references to colour in this figure legend, the reader is referred to the web version of this article.)

The elective treatment volume (CTV-3) will be personalized based on T-stage, midline extension and clinically involved levels according to Tables 1 and Supplementary Tables 1–5. Table 2 and Fig. 1 A-F provide examples of common de-escalation scenarios.

**Table 2:** Treatment recommendations for the most common presentations.

				**To be included in CTV-N**	**To be included in CTV-N**
**T-stage**	**Midline extension**	**Nodal involvement ipsilateral**	**Nodal involvement contralateral**	**Ipsilateral neck**	**Contralateral neck**
T1-4	FALSE	II	−	II, III	−
T1-4	TRUE	II	−	II, III	II
T1-2	FALSE	−	−	II	−
T3-4	FALSE	−	−	II, III	−
T1-2	TRUE	−	−	II	II
T3-4	TRUE	−	−	II, III	II
T1-4	FALSE	II, III	−	II, III, IVa	−
T1-4	TRUE	II, III	−	II, III, IVa	II
T1-2	TRUE	II	II	II, III	II, III
T3-4	TRUE	II	II	(Ib)*, II, III	II, III
T1-4	TRUE	II, III	II	II, III, IVa	II, III
T1-4	TRUE	II, III, IV	II	II, III, IV, V^**^	II, III
T1-4	FALSE	II, III	II	(Ib)*, II, III, IVa	II, III

*Only irradiate level IB electively if level IIA on the same side is involved.

**Level Va and Vb are irradiated.

Early T-stage is defined as T-stages 1–2 and advanced T-stage as T-stages 3–4. Midline extension is defined as a patient in whom any part of the primary tumor GTV extends beyond the midsagittal plane. Central tumors are those with similar tumor extension on both sides of the midsagittal plane by the judgement of the treating physician. Tumors extending on both sides but with the main tumor mass located on one side are instead classified as “lateralized tumors with midline extension”.

For defining clinical lymph node involvement, nodes with an uptake > 3 SUV or size > 1.5 cm or central necrosis on FDG-PET/CT are to be considered clinically involved unless confirmed negative through fine needle aspiration (FNA). In case of ambiguity after radiological work-up regarding lymph node involvement, FNA is recommended. We recommend FNA control of all ambiguous nodes > 1 cm, regardless of the SUV. For the definition of clinical lymph node involvement per level, in case of a lymph node conglomerate or large nodes extending over 2 levels, both levels should be considered as involved.

### Follow-up

During the treatment, there will be weekly clinical visits to evaluate toxicities. The first clinical assessment will take place 6–8 weeks after finishing treatment. An ultrasound examination of the neck LNLs is strongly recommended. Only nodes > 1 cm outside of the irradiated volume should be cytologically controlled with FNA within the first 4 months.

The first follow-up PET/T scan will take place 3–4 months after finishing treatment. Afterwards, follow-up visits will take place every 3 months during the first 2 years of follow-up, including assessment of toxicities and imaging if needed. If no imaging is performed, an ultrasound examination is mandatory to rule out lymph node recurrences/progression. Further standard follow-up PET/CT scans will take place 9–10 months and 2 years after finishing treatment. During the third year of follow-up, follow-up visits will take place every 6 months. If no imaging is performed, an ultrasound examination of the neck is mandatory.

### Endpoints

The primary endpoint is the rate of out-of-field N-site recurrences 2 years after the end of the primary (chemo)radiotherapy treatment. An event towards the primary endpoint is defined as any N-site recurrence in non-irradiated LNLs.

Secondary endpoints include the rate of out-of-field N-site recurrences 3 years after the end of the primary (chemo)radiotherapy treatment (in direct analogy to the primary endpoint), loco-regional control, progression-free survival and overall survival. For patients with recurrence, we will analyze detailed patterns of failure in terms of time to event and location of the recurrence, distinguishing T-site recurrence, N-site recurrence or persistence of grossly involved lymph nodes, isolated N-site recurrences in electively treated LNLs, and isolated recurrences in unirradiated LNLs. In addition, we will analyze early (≤3 months after treatment) and late (>3 months after treatment) toxicities of treatment: assessed by grading of toxicities according to CTCAE v5 [40]. Assessment of toxicities will be performed weekly (1x/week) during radiotherapy and during every follow-up visit. Overall toxicity will be evaluated according to the TAME methodology [41]. Other secondary endpoints include blood examination, including full blood count with lymphocytes taken during routine examination before and after the last radiotherapy, and quality of life (QoL) assessed using the EORTC QLQ C30 and Head and Neck Module HN43 questionnaires at the start of treatment, as well as after completing treatment, and 6, 12 and 24 months after radiotherapy [42], [43]. The evaluation of dysphagia using the M.D. Anderson Dysphagia Inventory (MDADI) [44] at 12 and 24 months is a planned addition.

Tumour response on imaging is assessed according to the RECIST criteria. Persistence is defined as progressive disease (PD), stable disease (SD) or incomplete response of the primary tumour (local persistence/persistence of the primary tumour) or nodal metastases at baseline (nodal persistence). Recurrence is defined as new pathological lesions/disease (new lesions or new lesions after complete response before) of the primary tumour (local recurrence), previous pathological lymph nodes (known lymph nodes), new lymph nodes, or distant metastases.

## Statistics

### Statistical methods

The primary goal of this study is to determine the rate of out-of-field N-site recurrences in nonirradiated lymph node levels after two years. The intervention is designed such that we expect a rate of out-of-field N-site recurrences in non-irradiated lymph node levels of < 10% (resulting in a value of 0.9 of the survival function at two years). The goal is to measure the cumulative probability of out-of-field recurrence with a precision resulting in a width of 10% of the 90% confidence interval of the Kaplan-Meier estimator at 2 years).

### Sample size calculation

120 patients will be included in this study. Under the assumption of no censoring or drop-outs, the intended width of 10% for the 90% confidence interval (from 0.84 to 0.94) for the survival probability of 0.9 can be obtained with 104 patients. By including 120 patients, we account for an amount of censoring or dropouts of 13%. The confidence interval was calculated for the log-minus-log transformation of the survival function using the asymptotic variance approximation as described in Nagashima et al. [45] and then back transformed.

### Statistical analyses

The primary endpoint will be evaluated according to the per-protocol treatment, although all primary analyses will follow both the per-protocol and intention-to-treat principles.

The primary endpoint “Out-of-field lymph node recurrence” will be reported as the Kaplan-Meier estimator for the time-to-event at 2 years. The calculation of the Kaplan-Meier estimator at 2 years will include the events detected at the 24-months follow-up visit (which may occur at 2 years +/- 1 month). Patients lost to follow-up and patients who die due to any cause are right-censored.

In addition to the overall rate of Out-of-field lymph node recurrences, we will analyze whether 1) Out-of-field lymph node recurrence was the initial cause for loco-regional failure, or whether 2) recurrence/persistence of the primary tumor or grossly involved lymph nodes present at baseline was observed simultaneously or prior to the Out-of-field lymph node recurrence. In case 1, irradiating the respective LNL electively may have prevented loco-regional failure. In case 2, loco-regional failure may have occurred irrespective of volume-de-escalation, and the Out-of-field lymph node recurrence may be due to lymphatic spread of the recurrent/persistent tumor. To determine the rate of “Out-of-field lymph node recurrences being the initial cause for loco-regional failure”, we will perform Kaplan-Meier analysis where patients with recurrence/persistence of the primary tumor or grossly involved lymph nodes present at baseline are right-censored.

### Safety interim analysis

If at any point in time during recruitment, the total number of events reported to the sponsor exceeds 20, no additional patients will be enrolled.

We will perform a safety interim analysis when 60 patients have been enrolled.

## Discussion

Our clinical trial aims to personalize the radiotherapy treatment of patients with oropharyngeal carcinoma. Our goal is to reduce treatment volume and herewith reduce radiotherapy-associated short-term and long-term side effects to improve patients’ quality of life. In our trial we test the hypothesis that the elective irradiation treatment volume can be personalized and reduced in most patients based on T-category and midline extension of the primary tumor and clinical involvement of the lymph nodes.

Previous de-escalation trials in squamous cell head and neck cancers, such as DIREKHT, EVADER, REWRITE and Van den Bosch et al., have shown promising locoregional control and overall survival rates, with improvement of toxicity [21], [22], [23], [46], [47]. DIREKHT included postoperative oral cavity, oropharynx, hypopharynx or larynx patients, and de-escalated treated volume, radiation dose, or both. Volume de-escalation consisted of omission of the contralateral elective nodal irradiation in case of contralateral pN0 or contralateral cN0 for lateralized tumors. Of the patients treated with reduced volume, 7.4% developed a locoregional recurrence. Four out of 143 patients (2.8%) that were treated with omission of the elective contralateral neck irradiation developed a regional recurrence in a contralateral neck node [21]. REWRITE de-escalated treatment for mainly laryngeal and oropharyngeal SCC patients by only including the LNLs directly adjacent to the primary tumor or involved LNLs. Two patients (3.6%) had a disease progression in the non-irradiated neck after after one year [22]. The phase III trial from Van den Bosch et al. included patients with oropharyngeal, hypopharyngeal and laryngeal SCC, and de-escalated treatment by reducing the dose to the elective neck to 35 Gy. The 2-year recurrence rate in electively irradiated nodes in the dose reduction groups was 4.9% compared to 4.3% in the control group [47], [48]. The phase II EVADER-trial resembles the DeEscO trial the most regarding de-escalation strategies but included only HPV-related oropharyngeal SCC patients. Here, volume-reduced ENI was tailored to primary location, T-category, and distribution of involves nodes. In total, five local recurrence events were observed, including one out-f-field regional control event [23].

HPV-related oropharyngeal SCC is associated with a better prognosis than patients with HPV-negative tumors [49]. Previous de-escalation efforts have distinguished de-escalation strategies between HPV-positive and HPV-negative patients [50]. Our trial instead includes patients irrespective of HPV-status with identical definitions of the CTV-N. The rationale is two-fold. First, the primary endpoint is N-site recurrences in unirradiated LNLs rather than locoregional control or overall survival. Second, based on our data on LNL involvement and the statistical model, we do not expect a substantial difference in the risk for occult metastases between HPV-positive and HPV-negative disease. Although HPV-positive tumors show a higher prevalence of lymph node involvement, especially for early T-category tumors, the probability of occult metastases conditioned on the clinical nodal status of an individual patient is similar according to our analysis.

A limitation of our trial is the lack of a control group. In part, the single-arm design is justified by the choice of the primary endpoint. Out-of-field N-site recurrence is an event specific to volume-de-escalation, which is expected to be very rare in a control group treated according to current ENI guidelines. Hence, the value of a control arm regarding the analysis of the primary endpoint is very limited. However, a randomized trial with a control group would have strengthened the analysis of secondary endpoints. Demonstrating toxicity reductions or non-inferiority in local control or overall survival may require a randomized trial. Because of the limited number of patients newly diagnosed with oropharyngeal cancer in Switzerland, this was considered outside the scope of this investigation.

In addition, the trial has limitations due to its pragmatic approach. Diagnostic procedures and follow-up are mostly limited to what is part of routine clinical care and we do not perform rigorous clinical trial QA during the trial. In addition, the trial protocol allows for small variations in institutional practice between the participating centers. For example, the use of an intermediate dose level in the PTV-2 is at the local investigator's discretion. However, given the well-defined dose prescriptions for PTV-1 and PTV-3, the dose fall-off around PTV- will likely be similar for patients planned with the intermediate dose and those treated without it. Hence, the addition of the intermediate dose is not expected to significantly impact dose distributions, toxicity, or quality of life.

Our research group is currently working on further improving the statistical models and extending the model to other tumor entities in the head and neck area, such as the oral cavity, hypopharynx, and larynx. Potentially, the personalization of the elective lymphatic irradiation volume could in the future also be extended to other tumor types with lymphatic spread patterns.

## Planned timeline

The first center opened in January 2025. Three other centers opened in February 2025. One other center opened in July 2025. The last center is planned to follow shortly afterwards. Recruitment is estimated to be completed by January 2027.

## Ethics committee approval

Ethical approval was obtained on 21/01/2025, BASEC-Nr 2024-01936. The study will be performed in accordance with the Declaration of Helsinki, the principles of Good Clinical Practice, the Human Research Act and the Human Research Ordinance, as well as other relevant local regulations.

## Consent for publication

Written informed consent is obtained from each participant prior to their inclusion in the study.

## Availability of data and materials

The previously collected multi-institutional dataset of 598 oropharyngeal SCC patients in whom the detailed patterns of lymph node involvement are reported is publicly available on https://www.LyProX.org. The data collected during the clinical trial will be published and be publicly available, after evaluation of each endpoint and concurrently with respective publications.

## Trial sponsor

Prof. Dr. med. Panagiotis Balermpas

Department of Radiation Oncology

University Hospital Zurich

Raemistrasse 100

CH 8091 ZURICH

Switzerland

E-Mail: Panagiotis.Balermpas@usz.ch

## Ethics approval and consent to participate

Ethical approval was obtained on 21/01/2025, BASEC-Nr 2024-01936. The study will be performed in accordance with the Declaration of Helsinki, the principles of Good Clinical Practice, the Human Research Act (HRA) and the Human Research Ordinance (HRO), as well as other relevant local regulations. Written informed consent is obtained from each participant prior to their inclusion in the study.

## Funding

The clinical trial will be funded internally. Preparation of the protocol and research leading to the design of the clinical trial was supported by the Clinical research priority program Artificial intelligence in oncological imaging of the University Zürich, and by the Swiss cancer research foundation under grant KFS-5645–08-2022.

## Declaration of Competing Interest

The authors declare that they have no known competing financial interests or personal relationships that could have appeared to influence the work reported in this paper.

## References

[1] Grégoire V, Ang K, Budach W, Grau C, Hamoir M, Langendijk JA, et al. Delineation of the neck node levels for head and neck tumors: a 2013 update. DAHANCA, EORTC, HKNPCSG, NCIC CTG, NCRI, RTOG, TROG consensus guidelines. Radiother Oncol. 2014;110(1):172–81. 10.1016/j.radonc.2013.10.010 24183870

[2] Grégoire V., Evans M., Le Q.T., Bourhis J., Budach V., Chen A. (2018). Delineation of the primary tumour Clinical Target Volumes (CTV-P) in laryngeal, hypopharyngeal, oropharyngeal and oral cavity squamous cell carcinoma: AIRO, CACA, DAHANCA, EORTC, GEORCC, GORTEC, HKNPCSG, HNCIG, IAG-KHT, LPRHHT, NCIC CTG, NCRI, NRG Oncology, PHNS, SBRT, SOMERA, SRO, SSHNO. TROG Consensus Guidelines Radiother Oncol.

[3] Biau J., Lapeyre M., Troussier I., Budach W., Giralt J., Grau C. (2019). Selection of lymph node target volumes for definitive head and neck radiation therapy: a 2019 Update. Radiother Oncol.

[4] Amin MB, Greene FL, Edge SB, Compton CC, Gershenwald JE, Brookland RK, et al. The Eighth Edition AJCC Cancer Staging Manual: Continuing to build a bridge from a population-based to a more “personalized” approach to cancer staging. CA Cancer J Clin. 2017;67(2):93–9. 10.3322/caac.21388 28094848

[5] Brierley J., Gospodarowicz M., Wittekind C. (2018).

[6] Machtay M., Moughan J., Trotti A., Garden A.S., Weber R.S., Cooper J.S. (2008). Factors associated with severe late toxicity after concurrent chemoradiation for locally advanced head and neck cancer: an RTOG analysis. J Clin Oncol.

[7] Shang J., Gu J., Han Q., Xu Y., Yu X., Wang K. (2014). Chemoradiotherapy is superior to radiotherapy alone after surgery in advanced squamous cell carcinoma of the head and neck: a systematic review and meta-analysis. Int J Clin Exp Med.

[8] Foster C.C., Seiwert T.Y., MacCracken E., Blair E.A., Agrawal N., Melotek J.M. (2020). Dose and volume de-escalation for human papillomavirus-positive oropharyngeal cancer is associated with favorable posttreatment functional outcomes. Int J Radiat Oncol Biol Phys.

[9] Price K., Van Abel K.M., Moore E.J., Patel S.H., Hinni M.L., Chintakuntlawar A.V. (2022). Long-term toxic effects, swallow function, and quality of life on MC1273: a phase 2 study of dose de-escalation for adjuvant chemoradiation in human papillomavirus-positive oropharyngeal cancer. Int J Radiat Oncol Biol Phys.

[10] Sher D.J., Pham N.L., Shah J.L., Sen N., Williams K.A., Subramaniam R.M. (2021). Prospective phase 2 study of radiation therapy dose and volume de-escalation for elective neck treatment of oropharyngeal and laryngeal cancer. Int J Radiat Oncol Biol Phys.

[11] Hegde J.V., Shaverdian N., Daly M.E., Felix C., Wong D.L., Rosove M.H. (2018). Patient-reported quality-of-life outcomes after de-escalated chemoradiation for human papillomavirus-positive oropharyngeal carcinoma: findings from a phase 2 trial. Cancer.

[12] Le Pechoux C., Pourel N., Barlesi F., Lerouge D., Antoni D., Lamezec B. (2022). Postoperative radiotherapy versus no postoperative radiotherapy in patients with completely resected non-small-cell lung cancer and proven mediastinal N2 involvement (lung ART): an open-label, randomised, phase 3 trial. Lancet Oncol.

[13] von der Grün J., Broglie M., Guckenberger M., Balermpas P. (2024). A comprehensive and longitudinal evaluation of the different populations of lymphoid and myeloid cells in the peripheral blood of patients treated with chemoradiotherapy for head and neck cancer. Cancer Immunol Immunother.

[14] Telarovic I., Yong C.S.M., Kurz L., Vetrugno I., Reichl S., Fernandez A.S. (2024). Delayed tumor-draining lymph node irradiation preserves the efficacy of combined radiotherapy and immune checkpoint blockade in models of metastatic disease. Nat Commun.

[15] Iorio G.C., Spieler B.O., Ricardi U., Dal Pra A. (2021). The impact of pelvic nodal radiotherapy on hematologic toxicity: a systematic review with focus on leukopenia, lymphopenia and future perspectives in prostate cancer treatment. Crit Rev Oncol Hematol.

[16] El Houat Y., Bouvier L., Baty M., Palard-Novello X., Pointreau Y., de Crevoisier R. (2022). Head and neck cancers volume reduction: should we reduce our prophylactic node radiation to spare the antitumor immune response?. Cancer Radiother.

[17] Morel D., Robert C., Paragios N., Grégoire V., Deutsch E. (2024). Translational frontiers and clinical opportunities of immunologically fitted radiotherapy. Clin Cancer Res.

[18] Leeman J.E., Li J.G., Pei X., Venigalla P., Zumsteg Z.S., Katsoulakis E. (2017). Patterns of treatment failure and postrecurrence outcomes among patients with locally advanced head and neck squamous cell carcinoma after chemoradiotherapy using modern radiation techniques. JAMA Oncol.

[19] van den Bosch S., Dijkema T., Verhoef L.C., Zwijnenburg E.M., Janssens G.O., Kaanders J.H. (2016). Patterns of recurrence in electively irradiated lymph node regions after definitive accelerated intensity modulated radiation therapy for head and neck squamous cell carcinoma. Int J Radiat Oncol Biol Phys.

[20] Martínez Carrillo M., Tovar Martín I., Martínez Lara I., de Almodóvar R., Rivera J.M. (2013). Del Moral Ávila R. Selective use of postoperative neck radiotherapy in oral cavity and oropharynx cancer: a prospective clinical study. Radiat Oncol.

[21] Haderlein M., von der Grün J., Balermpas P., Rödel C., Hautmann M.G., Steger F. (2024). De-intensification of postoperative radiotherapy in head and neck cancer irrespective of human papillomavirus status-results of a prospective multicenter phase II trial (DIREKHT Trial). Front Oncol.

[22] Castelli J., Sun X., Neveu E., Nguyen T.V., Tao Y., Martin L. (2024). 852MO REWRITE–GORTEC 2018-02: Radiotherapy-durvalumab without prophylactic neck irradiation in squamous cell carcinoma of the head and neck. Ann Oncol.

[23] Bratman S.V., Karam I., Waldron J.N., Butler J., Olson R.A., Pochini C.M. (2024). A phase II single arm trial of elective volume adjusted de-escalation radiotherapy (EVADER) in patients with low-risk HPV-related oropharyngeal squamous cell carcinoma. Int J Radiation Oncology*biology*physics.

[24] Kelly J.R., Husain Z.A., Burtness B. (2016). Treatment de-intensification strategies for head and neck cancer. Eur J Cancer.

[25] Woody N.M., Koyfman S.A., Xia P., Yu N., Shang Q., Adelstein D.J. (2016). Regional control is preserved after dose de-escalated radiotherapy to involved lymph nodes in HPV positive oropharyngeal cancer. Oral Oncol.

[26] Sanguineti G., Pai S., Agbahiwe H., Ricchetti F., Westra W., Sormani M.P. (2014). HPV-related oropharyngeal carcinoma with Overt Level II and/or III metastases at presentation: the risk of subclinical disease in ipsilateral levels IB. IV and v Acta Oncol.

[27] Kjems J., Gothelf A.B., Håkansson K., Specht L., Kristensen C.A., Friborg J. (2016). Elective nodal irradiation and patterns of failure in head and neck cancer after primary radiation therapy. Int J Radiat Oncol Biol Phys.

[28] Huang S.H., Waldron J., Bratman S.V., Su J., Kim J., Bayley A. (2017). Re-evaluation of Ipsilateral Radiation for T1-T2N0-N2b Tonsil Carcinoma at the Princess Margaret Hospital in the Human Papillomavirus Era, 25 Years later. Int J Radiat Oncol Biol Phys.

[29] Pouymayou B., Balermpas P., Riesterer O., Guckenberger M., Unkelbach J. (2019). A Bayesian network model of lymphatic tumor progression for personalized elective CTV definition in head and neck cancers. Phys Med Biol.

[30] Ludwig R., Pouymayou B., Balermpas P., Unkelbach J. (2021). A hidden Markov model for lymphatic tumor progression in the head and neck. Sci Rep.

[31] Ludwig R., Pérez Haas Y., Benavente S., Balermpas P., Unkelbach J. (2025). A probabilistic model of bilateral lymphatic spread in head and neck cancer. Sci Rep.

[32] Ludwig R., Schubert A.D., Barbatei D., Bauwens L., Hoffmann J.M., Werlen S. (2024). Modelling the lymphatic metastatic progression pathways of OPSCC from multi-institutional datasets. Sci Rep.

[33] Ludwig R., Hoffmann J.M., Pouymayou B., Däppen M.B., Morand G., Guckenberger M. (2022). Detailed patient-individual reporting of lymph node involvement in oropharyngeal squamous cell carcinoma with an online interface. Radiother Oncol.

[34] Ludwig R., Hoffmann J.M., Pouymayou B., Morand G., Däppen M.B., Guckenberger M. (2022). A dataset on patient-individual lymph node involvement in oropharyngeal squamous cell carcinoma. Data Brief.

[35] Ludwig R., Schubert A., Barbatei D., Bauwens L., Werlen S., Elicin O. (2024). A multi-centric dataset on patient-individual pathological lymph node involvement in head and neck squamous cell carcinoma. Data Brief.

[36] de Bondt R.B., Nelemans P.J., Hofman P.A., Casselman J.W., Kremer B., van Engelshoven J.M. (2007). Detection of lymph node metastases in head and neck cancer: a meta-analysis comparing US, USgFNAC, CT and MR imaging. Eur J Radiol.

[37] Ludwig R. (2023).

[38] Nutting C., Finneran L., Roe J., Sydenham M.A., Beasley M., Bhide S. (2023). Dysphagia-optimised intensity-modulated radiotherapy versus standard intensity-modulated radiotherapy in patients with head and neck cancer (DARS): a phase 3, multicentre, randomised, controlled trial. Lancet Oncol.

[39] Jensen K., Friborg J., Hansen C.R., Samsøe E., Johansen J., Andersen M. (2020). The Danish Head and Neck Cancer Group (DAHANCA) 2020 radiotherapy guidelines. Radiother Oncol.

[40] Common Terminology Criteria for Adverse Events (CTCAE) [Available from: https://www.eortc.be/services/doc/ctc/CTCAE_4.03_2010-06-14_QuickReference_5x7.pdf.

[41] Trotti A., Pajak T.F., Gwede C.K., Paulus R., Cooper J., Forastiere A. (2007). TAME: development of a new method for summarising adverse events of cancer treatment by the Radiation Therapy Oncology Group. Lancet Oncol.

[42] EORTC Quality of Life of Cancer Patients Questionnaire [Available from: https://www.eortc.org/app/uploads/sites/2/2018/08/Specimen-QLQ-C30-English.pdf.

[43] EORTC Head & Neck Cancer (update of QLQ-H&N35) [Available from: https://www.eortc.org/app/uploads/sites/2/2018/08/Specimen-HN43-English.pdf.

[44] Chen A.Y., Frankowski R., Bishop-Leone J., Hebert T., Leyk S., Lewin J. (2001). The development and validation of a dysphagia-specific quality-of-life questionnaire for patients with head and neck cancer: the M. D. Anderson dysphagia inventory. Arch Otolaryngol Head Neck Surg.

[45] Nagashima K., Noma H., Sato Y., Gosho M. (2021). Sample size calculations for single-arm survival studies using transformations of the Kaplan-Meier estimator. Pharm Stat.

[46] Reichl S., Pruschy M., Kreuzer M., Balermpas P., Telarovic I. (2025). Translating spared and delayed tumor-draining lymph node irradiation into clinical practice: current clinical trials and future directions. Eur J Cancer.

[47] van den Bosch S., Dijkema T., Kunze-Busch M.C., Terhaard C.H., Raaijmakers C.P., Doornaert P.A. (2017). Uniform FDG-PET guided GRAdient Dose prEscription to reduce late Radiation Toxicity (UPGRADE-RT): study protocol for a randomized clinical trial with dose reduction to the elective neck in head and neck squamous cell carcinoma. BMC Cancer.

[48] van den Bosch S., Doornaert P.A.H., Hoebers F.J.P., Kreike B., Vergeer M.R., Zwijnenburg E.M. (2025). Clinical Benefit and Safety of Reduced Elective Dose in Definitive Radiotherapy for Head and Neck Squamous Cell Carcinoma: the UPGRADE-RT Multicenter Randomized Controlled Trial. J Clin Oncol.

[49] Ang K.K., Harris J., Wheeler R., Weber R., Rosenthal D.I., Nguyen-Tân P.F. (2010). Human papillomavirus and survival of patients with oropharyngeal cancer. N Engl J Med.

[50] Petrelli F., Luciani A., Ghidini A., Cherri S., Gamba P., Maddalo M. (2022). Treatment de-escalation for HPV+ oropharyngeal cancer: a systematic review and meta-analysis. Head Neck.

