# Supplementary material for: Personalized volume de-escalated elective nodal irradiation in oropharyngeal squamous cell carcinoma (DeEscO): a study protocol
Source: Clin Transl Radiat Oncol. 2026 May 12;59:101182. doi: 10.1016/j.ctro.2026.101182 (PMC13199938; doi:10.1016/j.ctro.2026.101182)
Supplement: Supplementary Data 1 [file mmc1.docx]

**Supplementary Table 1. Treatment recommendations for central tumors or tumors without clear lateralization.**

|  |  |  | **To be included in CTV-N** | **To be included in CTV-N** |
| --- | --- | --- | --- | --- |
| **T-stage** | **Nodal involvement side one** | **Nodal involvement side two** | **Side one neck** | **Side two neck** |
| T1-2 | - | - | II | II |
| T1-2 | II | - | II, III | II, III |
| T1-2 | III | - | II, III, IVa | II, III |
| T1-2 | II, III | - | II, III, IVa | II, III |
| T1-2 | II | II | (Ib)*, II, III | (Ib)*, II, III |
| T1-2 | II | III | (Ib)*,II, III | II, III, IVa |
| T1-2 | III | III | II, III, IVa | II, III, IVa |
| T1-2 | II, III | II | (Ib)*, II, III, IVa | II, III |
| T1-2 | II, III | III | (Ib)*, II, III, IVa | II, III, IVa |
| T1-2 | II, III | II, III | (Ib)*, II, III, IVa | (Ib)*, II, III, IVa |
| T3-4 | - | - | II, III | II, III |
| T3-4 | II | - | II, III | II, III |
| T3-4 | III | - | II, III, IVa | II, III |
| T3-4 | II | II | (Ib)*, II, III | (Ib)*, II, III |
| T3-4 | II | III | (Ib)*, II, III | II, III, IVa |
| T3-4 | III | III | II, III, IVa | II, III, IVa |
| T3-4 | II, III | - | (Ib)*, II, III, IVa | II, III |
| T3-4 | II, III | II | (Ib)*, II, III, IVa | (Ib)*, II, III |
| T3-4 | II, III | III | (Ib)*, II, III, IVa | II, III, IVa |
| T3-4 | II, III | II, III | (Ib)*, II, III, IVa | (Ib)*, II, III, IVa |

*Only irradiate level IB electively if level IIA on the same side is involved.

**Supplementary Table 2. Treatment recommendations for lateralized tumors, with midline extension and early T-stage (T1-2).**

|  |  | **To be included in CTV-N** | **To be included in CTV-N** |
| --- | --- | --- | --- |
| **Nodal involvement ipsilateral** | **Nodal involvement contralateral** | **Ipsilateral Neck** | **Contralateral Neck** |
| - | - | II | II |
| II | - | II, III | II |
| III | - | II, III, IVa | II |
| VII | - | II, III, VII | II |
| II, III | - | II, III, IVa | II |
| II, VII | - | II, III, VII | II |
| III, VII | - | II, III, IVa, VII | II |
| II, III, IV | - | II, III, IV, V** | II |
| II | II | II, III | II, III |
| II, III | II | II, III, IVa | II, III |
| II, III, IV | II | II, III, IV, V** | II, III |
| II, III | II, III | (Ib)*, II, III, IVa | II, III, IVa |

* Only irradiate level IB electively if level IIA on the same side is involved.
** Level Va and Vb are irradiated.

**Supplementary Table 3. Treatment recommendations for lateralized tumors, with midline extension and advanced T-stage (T3-4).**

|  |  | **To be included in CTV-N** | **To be included in CTV-N** |
| --- | --- | --- | --- |
| **Nodal involvement ipsilateral** | **Nodal involvement contralateral** | **Ipsilateral Neck** | **Contralateral Neck** |
| - | - | II, III | II |
| II | - | II, III | II |
| III | - | II, III, IVa | II |
| II, III | - | II, III, IVa | II |
| II, VII | - | II, III, VII | II |
| Ib, II, III | - | Ib, II, III, IVa | II |
| II, III, IV | - | II, III, IV, V** | II |
| II, III, IV, V | - | (Ib)*, II, III, IV, V** | II |
| II | II | (Ib)*, II, III | II, III |
| II, III | II | (Ib)*, II, III, IVa | II, III |
| II, VII | II | II, III, VII | II, III |
| Ib, II, III | II | Ib, II, III, IVa | II, III |
| II, III, IV | II | II, III, IV, V** | II, III |
| II, III, VII | II | II, III, IVa, VII | II, III |
| II | II, III | (Ib)*, II, III | II, III, IVa |
| II, III, IV | II, III | II, III, IVa, V** | II, III, IVa |
| II, III | II, III | (Ib)*, II, III, IVa | II, III, IVa |
| II, III | II, III, IV | (Ib)*, II, III, IVa | II, III, IV, V** |

* Only irradiate level IB electively if level IIA on the same side is involved.
** Level Va and Vb are irradiated. Level Vc is irradiated if level Vb is involved.

**Supplementary Table 4. Treatment recommendations for lateralized tumors, without midline extension and early T-stage (T1-2).**

|  |  | **To be included in CTV-N** | **To be included in CTV-N** |
| --- | --- | --- | --- |
| **Nodal involvement ipsilateral** | **Nodal involvement contralateral** | **Ipsilateral Neck** | **Contralateral Neck** |
| - | - | II | - |
| II | - | II, III | - |
| III | - | II, III, IVa | - |
| IV | - | II, III, IV, V** | - |
| V | - | II, III, IVa, V** | - |
| Ib, II | - | Ib, II, III | - |
| II, III | - | II, III, IVa | - |
| II, V | - | II, III, IVa, V** | - |
| II, VII | - | II, III, VII | - |
| Ib, II, III | - | Ib, II, III, IVa |  |
| II, III, IV | - | II, III, IV, V** | - |
| II, III, V | - | II, III, IVa, V** | - |
| II, III, VII | - | II, III, IVa, VII | - |
| II, III, IV, V | - | II, III, IV, V** | - |
| II | II | II, III | II, III |
| II, III | II | (Ib)*, II, III, IVa | II, III |
| II, III, IV | II | II, III, IV, V** | II, III |

* Only irradiate level IB electively if level IIA on the same side is involved.
** Level Va and Vb are irradiated. Level Vc is irradiated if level Vb is involved.

**Supplementary Table 5. Treatment recommendations for lateralized tumors, without midline extension and advanced T-stage (T3-4).**

|  |  | **To be included in CTV-N** | **To be included in CTV-N** |
| --- | --- | --- | --- |
| **Nodal involvement ipsilateral** | **Nodal involvement contralateral** | **Ipsilateral Neck** | **Contralateral Neck** |
| - | - | II, III | - |
| II | - | II, III | - |
| III | - | II, III, IVa | - |
| IV | - | II, III, IV, V** | - |
| Ib, II | - | Ib, II, III | - |
| II, III | - | II, III, IVa | - |
| II, V | - | II, III, IVa, V** | - |
| Ib, II, III | - | Ib, II, III, IVa | - |
| II, III, IV | - | II, III, IV, V** | - |
| II, III, V | - | II, III, IVa, V** | - |
| II | II | (Ib)*, II, III | II, III |
| II, III | II | (Ib)*, II, III, IVa | II, III |
| II, III, IV | II | (Ib)*, II, III, IV, V** | II, III |

* Only irradiate level IB electively if level IIA on the same side is involved.

** Level Va and Vb are irradiated. Level Vc is irradiated if level Vb is involved.
